# Focus on Nutritional Aspects of Sarcopenia in Diabetes: Current Evidence and Remarks for Future Research

**DOI:** 10.3390/nu14020312

**Published:** 2022-01-13

**Authors:** Christian Göbl, Andrea Tura

**Affiliations:** 1 Department of Obstetrics and Gynaecology, Medical University of Vienna, 1090 Vienna, Austria; christian.goebl@meduniwien.ac.at; 2 CNR Institute of Neuroscience, 35127 Padova, Italy

## Sarcopenia and Diabetes: Basic Information

Sarcopenia has been defined as a progressive and generalized loss of muscle mass that can be observed after the age of 40 years, with a rate of deterioration of about 8% every ten years up to 70 years, and 15–25% thereafter [1]. Of note, it was reported that the prevalence of sarcopenia worldwide ranges from 10% up to 40%, depending on the criteria used to assess the disease and on the different characteristics of the population (such as ethnicity and other comorbidities or conditions of frailty) [2]. As an example, sarcopenia often coexists with obesity (due to aging, reduction in physical activity, malnutrition, sub-clinical inflammation, and hormonal changes), and hence the term “sarcopenic obesity” has been introduced [3]. In recent years, attention has also been increased for the coexistence of sarcopenia with another common chronic disease, i.e., type 2 diabetes (T2DM) [4]. In fact, sarcopenia, and frailty in general, is emerging as a further category of complications in T2DM, in addition to those already well known, such as micro and macrovascular diseases [5].

## Nutritional Aspects in Sarcopenia and Diabetes: Main Concepts from Review and Meta-Analysis Studies

An interesting review article, published in *Nutrients* in January 2021 by Izzo A. et al. [6], has provided an overview on the association between sarcopenia and several features of T2DM, with special attention given to aging, overweight/obesity, sex, T2DM duration, use of antidiabetic medications, presence of T2DM complications, and nutritional status. The aim of this editorial is to summarize the main nutritional aspects of relevance in the presence of sarcopenia and T2DM and to provide an update on the most recent findings in this field.

In the *Nutrients* review [6], it was reported that a high prevalence of sarcopenia has been observed in T2DM subjects with poor nutritional status [7]. More precisely, one study reported that in T2DM subjects with sarcopenia the energy intake was significantly lower than in those without sarcopenia, whereas no difference in the intake of proteins, carbohydrates, or fats was observed [8]. In another review [9], it is also suggested that elderly people may suffer from inadequate protein intake, this yielding to impaired sustainment of the muscle mass and strength [10]. In addition, an inappropriate energy intake may cause a reduction in the muscle protein synthesis, even when the supply of proteins is within a normal range [11]. On the other hand, surprisingly, a meta-analysis [12] revealed that the prevalence of sarcopenia in T2DM was not significantly associated with the dietary protein intake. It is also worth noting that in sarcopenic obesity the determination of the optimal energy intake is challenging, since some degree of caloric restriction may be beneficial, but exaggerated restriction may determine the worsening of sarcopenia, for the reasons explained. Thus, some studies suggested inappropriateness of acute (strong) caloric restriction, whereas chronic (moderate) caloric restriction may allow an increase rather than downregulation in muscle protein synthesis, especially when accompanied by a sufficiently high protein intake [13,14,15,16]. Protein supplement should also be considered when adequately high dietary protein intake is not feasible [9]. In addition, it should be mentioned that there are indications that the Mediterranean diet, especially regarding the high consumption of fruits and vegetables [6], rather than high-protein diets, is beneficial for those with sarcopenia.

Besides proteins, in the *Nutrients* review [6] it is also reported that in T2DM subjects with sarcopenia omega-3 fatty acids intake has been found to be reduced compared with those without sarcopenia [8]. Indeed, omega-3 fatty acids may act to reduce inflammation (thus improving the strength of the skeletal muscle) [17], stimulating muscle protein synthesis [18] and improving overall the neuromuscular function [19]. Vitamin D may also be beneficial for those suffering sarcopenia, as discussed in another review study [20], since vitamin D may improve both muscle mass and function. However, the *Nutrients* review [6] reports that the optimal dose and the frequency of doses are still unclear, as well as the treatment duration, to reach such goals. Of note, other vitamins may play a role in sarcopenia, especially vitamin C, B6 and B12, A, and E [20]. For those with sarcopenia, branched-chain amino acids supplementation (especially leucine) may be beneficial as well [21]. Selenium and magnesium supplements may also be useful, as they appear to show an association with physical activity, muscle performance, and bone health in elderly people in general [22]. Other micronutrients with possible effects in sarcopenia are calcium, potassium, phosphorus, iron, and zinc [20].

The review [6] also reports that there are no differences in the prevalence of alcohol consumption between T2DM subjects with and without sarcopenia [23,24,25,26]. Among the drinking habits, it was also reported that regular consumption of coffee has a protective role against the development of sarcopenia [27,28], likely due to coffee’s anti-inflammatory and antioxidant properties [29]. On the other hand, coffee consumption appears to be associated with a lower risk of T2DM [30,31]. Tea consumption may also be beneficial for those with sarcopenia; specifically, green tea polyphenols and catechins have been shown to exhibit antioxidant effects [32,33].

## Recent Evidence about Relationship between Nutrition, Sarcopenia and Diabetes

What other articles have been published in the last few months focusing on nutritional aspects in sarcopenia and diabetes (which have not been reviewed in the *Nutrients* article [6])? Some articles further explored the aspect of the relationship between malnutrition and sarcopenia. One study evaluated some metabolic characteristics (including parameters of glucose metabolism) in addition to the nutritional status of both middle-aged and elderly subjects with sarcopenia and T2DM [34]. Subjects were grouped into a probable sarcopenia group (low muscle strength, *n* = 405) and a non-sarcopenia control group (normal muscle strength, *n* = 720). The probable sarcopenia subjects were older and had lower waist-to-hip ratios and BMIs, a longer diabetes duration, higher fasting plasma glucose level and glycosylated hemoglobin, decreased estimated glomerular filtration rate, lower bone mineral content, and lower fatless upper arm circumference, appendicular skeletal muscle mass index, and muscle quality. All subjects recorded a diet diary for three continuous days in accordance with appropriate nutrition counseling, with the registration of the weight of each type of food. Calculations were then performed to derive the total energy, carbohydrate, protein, and fat intake per day, and the proportion of calories supplied by the three macronutrients, with adjustments for body weight as appropriate. When comparing the probable sarcopenia and control groups, in the former the calorie percentage supplied by carbohydrates was found to be lower, while the percentage supplied by fat was higher, though the difference was significant only in men. In addition, some parameters that may be markers of malnutrition were found to be impaired in the probable sarcopenia group (lower hemoglobin, hematocrit, and albumin levels in men and the fatless upper arm circumference in both sexes).

In another study [35], nutrition counseling was evaluated retrospectively in subjects with T2DM to assess its possible effect in preventing sarcopenia. Nutrition counseling information was derived from medical records for a long period (2 years, rather than the typical 3–6-month duration of other studies in the same area). A group of 1164 subjects was included in the analysis for the counseling evaluation. Medical records showed that approximately one-third of the subjects received no counseling, but about one-third had at least three out of a maximum of ten sessions. In addition, the attitude towards nutrition counseling was assessed by the subjects’ responses during the counseling sessions, summarizing this into a three-level categorical variable. It was found that more frequent counseling sessions were associated with a lower risk of sarcopenia regardless of age, body weight, diabetes duration, or quality of the glycemic control.

Another approach was assessing the association of malnutrition with sarcopenia in subjects with T2DM by exploiting different definitions of malnutrition, essentially based on anthropometric, phenotypic, and etiological parameters [36]. Specifically, the guidelines considered for the assessment of malnutrition were those of the European Society for Clinical Nutrition and Metabolism (ESPEN) and those of the Global Leadership Initiative on Malnutrition (GLIM). In addition, the Subjective Global Assessment (SGA) criteria were considered. The ESPEN criteria include age, BMI, and the possible degree of unintentional weight loss in a given time interval. The GLIM criteria include the appropriate combination of etiological and phenotypic parameters among age-dependent BMI and unintentional weight loss (these being similar to the ESPEN criteria), plus insufficient energy intake or its reduction at any rate higher than a prescribed threshold in a given time interval; the presence of chronic diseases and inflammatory conditions are also considered. The SGA criteria are more complex, also including anamnestic parameters related, for instance, to variations in nutrients intake and the presence of gastrointestinal symptoms [37]. It was found that in the analyzed T2DM population (311 subjects with age ≥ 60 years) it was possible to predict sarcopenia through all the considered malnutrition criteria, i.e., ESPEN GLIM, and SGA. Mid-arm circumference and adductor pollicis muscle thickness (APMT) were also measured and were found to have a role in sarcopenia prediction. On the other hand, it appears that SGA criteria plus APMT measurements may be better predictors of sarcopenia in older people with T2DM, compared to the other analyzed malnutrition criteria and measures.

The geriatric nutritional risk index (GNRI), rather than the ESPEN, GLIM, or SGA criteria, was considered in another study [38]. Compared to other indices or criteria GNRI appears simple, but with the peculiar aspect of considering the serum albumin level, in addition to current and ideal body weight (the latter estimated from the subject’s height and a reference BMI of 22 kg/m^2^). In total, 526 subjects with T2DM were analyzed, and 452 of them also completed a survey about their dietary habits in the last month, allowing calculation of total energy per day, as well as total protein, fat, and carbohydrate intakes. It was found that low GNRI is related to the prevalence of sarcopenia. Consistently, the proportion of sarcopenia in participants with low GNRI was higher than that with high GNRI. However, there was no difference of dietary habits between the high- and the low-GNRI groups.

One study [39] analyzed sarcopenia in subjects that underwent kidney transplantation, who also suffered from T2DM in 22% of cases (precisely, 25% in the non-sarcopenia subjects, *n* = 186 and 17% in the sarcopenia subjects, *n* = 24). A peculiar aspect of this study was considering, in addition to BMI, the phase angle parameter (PhA), derived by the reactance and resistance of the subject’s body as obtained from bioelectrical impedance measurements, as a further possible marker of the nutritional status, which could possibly be useful for the detection of sarcopenia. In the whole population, it was found that PhA and BMI were negatively correlated with sarcopenia, after adjusting for several variables such as age, sex, dialysis vintage, time after transplant, presence of diabetes, hemoglobin, and estimated glomerular filtration rate. Of note, based on a receiver–operating characteristic curve analysis, the optimal cutoff values for both BMI and PhA were suggested for sarcopenia detection in the studied type of subjects.

Other recent studies were not focused on the evaluation of the dietary habits and possible malnutrition status but rather on the effects of some specific macro or micronutrients, some of which may have beneficial effects on sarcopenia in T2DM. One article [40] noted that no precise dietary recommendations exist specifically for skeletal muscle health during ageing, which may be crucial to preventing sarcopenia, especially in the presence of T2DM. It was also highlighted that some studies indicate a high total fat and saturate fatty acids (SFA) intake is detrimental to skeletal muscle, while higher intakes of polyunsaturated fatty acids (PUFA) appear to be protective. However, it was reported that the recent report of the Scientific Advisory Committee on Nutrition on SFA and health, published in 2019, made no specific comments on the effects of SFA on the skeletal muscle, due to insufficient evidence.

One review article [41] addressed the possible effects of some nutritional substrates involved in muscle synthesis, i.e., leucine (Leu), glutamine (Gln), arginine (Arg), and hydroxyl-methyl butyrate (HMB), which is an active metabolic form of leucine. It was concluded that there is evidence suggesting that the addition of dietary supplements enriched in certain amino acids leads to improvements in muscle strength and mass. More precisely, connections among amino acid supplementation, sarcopenia, and T2DM prevention have been supported by the literature, and evidence was reported for the beneficial effects of a dietary supplement based on Leu, Gln, Arg, and HMB. In more details, HMB and Leu have shown their potential for sarcopenia treatment, due to their ability to increase muscle protein synthesis. HMB has also been reported to have upregulated function when paired with Arg and Gln. Arg may not prevent muscle deterioration in isolation, but when combined with HMB and Leu its beneficial effect seems to emerge. Similar results have also been obtained with Gln alone and in combination with other amino acids.

Another review article [42] revised the available evidence about the effects of nutraceuticals for the prevention or the treatment of sarcopenia. The article focused on bee products (royal jelly, propolis, and bee pollen), which are rich in potent antioxidants such as flavonoids, phenols, and amino acids. It was reported that these bee products have been used to treat multiple chronic conditions predisposed to muscle wasting, such as cardiovascular diseases and diabetes, thanks to their rich and differentiated pharmacological activities (e.g., antioxidant, anti-inflammatory, antimicrobial, anti-allergic, anti-aging, anticarcinogenic, etc.). The article concludes that data from reviewed studies collectively denote varying levels of positive effects of bee products on muscle mass, strength, and function, the underlying mechanisms likely including amelioration of inflammation and oxidative damages, promotion of metabolic regulation, enhancement of stem cell responsiveness, improvement of muscular blood supply, inhibition of catabolic genes, and promotion of peripheral neuronal regeneration.

One article [43] described a randomized, double-blind, placebo-controlled trial aimed at investigating the effects of Korean Red Ginseng (KRG) on biomarkers of sarcopenia in middle-aged and elderly subjects with T2DM. Fifty-nine patients were randomly allocated to either placebo or KRG and took corresponding tablets for 24 weeks. The primary study outcomes were changes in sarcopenia biomarkers at week 24, namely follistatin (a mediator of muscle synthesis) and sex hormone-binding globulin (SHBG), which plays an important role in the synthesis and maintenance of muscle in relation to the growth hormone. It was found that the administration of KRG in diabetes patients for the indicated period resulted in a significant improvement in both follistatin and SHBG levels, especially in postmenopausal women (age > 55 years).

Finally, one article [44] was published on the possible beneficial effect of one traditional Japanese dish, i.e., miso soup, for those with sarcopenia related to T2DM. Such a soup is made from a fermented soybean food called miso, and it includes vitamins, minerals, vegetable proteins, microorganisms, salts, carbohydrates, and fat [45]. In the study [44], 192 men and 159 women with T2DM were included. It was found that habitual miso consumption was inversely associated with the presence of sarcopenia in women but not in men.

## Summary and Concluding Comments

This editorial started with a review article recently published in *Nutrients* [6]. We first summarized the main aspects of nutrition related to sarcopenia and T2DM, as revised in the review article [6]. Afterwards, we also considered some further reviews and then moved to the analysis of articles published in the last few months that were not included in the *Nutrients* review [6]. Based on our analysis, we can claim that some aspects of nutrition in relation to sarcopenia in T2DM deserve further investigation. Possible directions for future research are suggested in some of the articles mentioned in this editorial. The article that focused on fatty acids [40] reported that to date there are no dietary recommendations specifically tailored for maintaining skeletal muscle health during ageing, and this may be relevant to preventing or delaying the onset of sarcopenia or their detrimental consequences, especially in T2DM. In the review article focused on amino acids [41], it was concluded that no individual amino acid has been found to be sufficiently effective in preventing muscle deterioration, sarcopenia, or ultimately T2DM, but when appropriately combined the above-mentioned amino acids may stimulate an increase in muscle mass and strength in sarcopenic elderly individuals, possibly also decreasing T2DM risk. However, the optimal dosing in an ideal amino acids mixture and treatment duration remain to be elucidated in future studies. The review article [42] emphasizes the importance of future studies addressing the effect of individual variability (e.g., sex, general health, activity level, diet habits) on the effectiveness of nutraceuticals (such as the bee products, the focus of the review) that appear relevant for muscle health and hence for sarcopenia prevention or treatment. We in fact agree with this position since there is increasing evidence of the importance of precision medicine in advancing the treatment of several diseases and for the general wellness, especially that of elderly people. This is certainly valid even in the field of metabolism and nutrition, as outlined, for instance, in a recent consensus report regarding precision medicine in diabetes [46]. Another direction for future research in our opinion may be investigating relationships between sarcopenia and other types of diabetes, especially type 1 diabetes. Indeed, we suspect that there may be aspects, including some related to nutrition, that could be different compared to type 2 diabetes. To our knowledge, to date few studies have focused on sarcopenia and type 1 diabetes in humans [47,48], and none reported longitudinal data. In summary, sarcopenia in diabetes appears to be a research field still requiring relevant investigation effort from several viewpoints, including those specifically related to nutrition.
